# Medical Education

**Published:** 1857-10

**Authors:** 


*Medical Education.*

We are glad to see that many of our exchanges are still discussing this trite topic. The *Virginia Medical Journal* is advocating a plan for uniting the two Medical Colleges in that state, adding one-third to the number of Professorships, and greatly extending the length of the college term. The same Journal also strongly advocates the doctrine, that each section of the country should educate its own physicians. Let the discussion go on, and we have full faith that correct principles will be evolved, and ultimately acted on.

